# Effects of *Bacillus subtilis* and *Rhodotorula* Yeast Culture on the Growth Performance, Meat Quality, Antioxidant Capacity, and Serum Metabolites in Yellow-Feathered Broilers

**DOI:** 10.3390/biology14070820

**Published:** 2025-07-05

**Authors:** Ke Wang, Xiangtan Su, Xinyu Lu, Guang Yang, Gaowei Zhang, Jiwei Chen, Jiale Sun, Aiqin Gao

**Affiliations:** College of Animal Science, Inner Mongolia Agricultural University, Hohhot 010018, China

**Keywords:** *Bacillus subtilis*, *Rhodotorula* yeast culture, meat quality, serum metabolites, yellow-feathered broilers

## Abstract

This study aimed to investigate the effects of dietary supplementation with *Bacillus subtilis* (BS) and *Rhodotorula* yeast culture (RYC) used as feed additives to study their effects on growth performance, meat quality, antioxidant capacity, and serum metabolomics in yellow-feathered broilers. A total of 192 one-day-old healthy yellow-feathered broilers were randomly divided into four treatment groups. The CON group was fed a basal diet, whereas the BS, RYC, and MIX groups were fed diets supplemented with 5 × 10^9^ CFU/kg BS, 5000 mg/kg RYC, and a combination of both, respectively. The experimental period comprised 56 days. In the initial stage of growth, compared with the CON group, the body weight and average daily gain of broilers in the BS group were significantly increased (*p* < 0.05). The BS group increased the activity of CAT and SOD in broilers at 28 days, and the activity of CAT in RYC and MIX group were also significantly increased (*p* < 0.05). BS could increase GSH-Px activity significantly in broilers at 56 days (*p* < 0.05). Moreover, in comparison with the CON group, the MIX group decreased shear force and drip loss significantly (*p* < 0.05). Serum metabolome analysis showed that the addition of BS or RYC alone had the greatest difference in the abundance of D-amino acid metabolism and lysine metabolism, while the combined addition of BS and RYC found that glycerophospholipid metabolism and tryptophan metabolism had the greatest difference. In conclusion, dietary addition of BS and RYC could improve the meat quality of yellow-feathered broilers.

## Simple Summary

Yellow-feathered broilers, a distinctive breed in China, are favored by consumers because of their delicious meat and rich nutrition. However, in the past, the focus of industrial development was only on intensive breeding to achieve faster growth. With the improvement of consumers’ living standards, more and more people are beginning to care about the quality of broilers. Therefore, how to improve the meat quality of yellow-feathered broilers has become a new research hotspot. In this experiment, *Bacillus subtilis* (BS) and *Rhodotorula* yeast culture (RYC) were used as feed additives to study their effects on growth performance, meat quality, and antioxidant capacity of yellow-feathered broilers. Previous studies have established that BS confers health benefits in animals through oxidative stress mitigation, while RYC exhibits significant potential as an antioxidant source. The results demonstrated that the simultaneous addition of BS and RYC improved the meat quality and antioxidant capacity of yellow-feathered broilers. Metabolomic profiling revealed that both BS and RYC significantly modulated amino acid metabolic pathways, demonstrating considerable potential for practical applications in animal nutrition.

## 1. Introduction

Yellow-feathered broilers, a characteristic breed with a long history in China, have become a favorite food due to their mellow meat flavor and delicate taste [1]. However, traditional poultry industry development has predominantly focused on accelerated growth rates through intensive breeding systems to achieve shorter production cycles [2]. With the improvement of living standards, consumers are increasingly concerned about the quality of broilers. More and more research findings indicate that dietary intervention is one of the effective measures to improve broiler quality [3,4]. Therefore, finding an effective feed additive to enhance the meat quality of yellow-feathered broilers has become an urgent concern in the Chinese broiler industry.

Probiotics, as a group of bioactive microbial preparations with well-documented beneficial effects, have been widely utilized in livestock and poultry production, demonstrating positive impacts on enhancing animal growth performance and improving antioxidant capacity [5]. Among various probiotic strains, *Bacillus subtilis* (BS) represents a highly characteristic probiotic that has been demonstrated to secrete multiple bioactive enzymes, enhance nutrient utilization in host organisms, and consequently improve both health status and production performance in livestock and poultry [6]. Research has shown that dietary supplementation with BS at 3 × 10^9^ CFU/g significantly increased carcass weight, enhanced glutathione peroxidase (GSH-Px) activity in pectoral muscle tissue, and improved both redness and tenderness in Arbor Acres (AA) broilers [7]. The research conducted by Li et al. [8] furnished further evidence that the inclusion of 10^8^ CFU/kg of *Bacillus subtilis* powder in the basal diet can lead to an increase in average daily gain (ADG) and final body weight in Ross 308 broilers, alongside an improvement in serum antioxidant capacity. Liu et al. [9] showed that adding 5 × 10^8^ CFU/kg *Bacillus subtilis* HC6 to the diet could significantly improve the feed conversion rate of white feather broilers and improve the antioxidant capacity in serum and liver tissue. Research findings have confirmed that the dietary supplementation with a BS preparation at a spore concentration of 2.0 ×10^9^ spores/g could reduce drip and cooking loss in AA broilers, and improve the compositional ratios of essential amino acids and polyunsaturated fatty acids [10]. The aforementioned studies collectively indicate that they exhibit significant potential for improving meat quality parameters in poultry production.

Yeast culture (YC) is a kind of yeast additive, which is mainly rich in protein, β-glucan, mannan oligosaccharides, peptides, yeast cell metabolites, and digestive enzymes [11]. Several studies have demonstrated that supplementing feed with yeast culture (YC) confers multiple beneficial effects on livestock and poultry, including improved growth performance, enhanced serum antioxidant enzyme activity, reduced lipid peroxidation damage, and improved meat quality traits [12,13,14,15]. Fungal species of the genus *Rhodotorula*, such as *R. mucilaginosa* and *R. minuta*, are known for their broad distribution, which has the characteristics of producing active metabolites such as carotenoids, digestive enzymes, β-glucan, and vitamins [16,17]. Existing studies have shown that *Rhodotorula mucilaginosa* C2.5t1 could offset the pro-oxidation effect of hydrogen peroxide by regulating carotenoid content [18]. It was also found that oral administration of *R.mucilaginosa* TZR_2014_ improved the growth performance and antioxidant capacity of piglets [17]. Recent studies have developed a *Rhodotorula mucilaginosa* solid-state fermentation product (RSFP) and demonstrated that dietary supplementation with 0.5%, 2.5%, or 12.5% RSFP in basal diets significantly enhanced yolk color and improved intestinal health parameters in Roman laying hens [19]. Our preliminary research findings indicated that *Rhodotorula* Yeast Culture (RYC), derived from *Rhodotorula* species, exerted positive effects on animals. Specifically, dietary supplementation with RYC improved mutton quality and enhanced its antioxidant capacity [20]. Therefore, the RYC has indeed demonstrated the ability to improve the quality of livestock and poultry meat and to enhance the body’s antioxidant capacity.

Current research indicates that BS has the potential to enhance the growth performance and meat quality of poultry, and the antioxidant properties of RYC may also have a positive effect on the meat quality of livestock products. Based on this, we hypothesize that the combined use of BS and RYC could more effectively improve meat quality. However, there is a lack of studies on the integrated application of BS and RYC. Therefore, this study utilized yellow-feathered broilers as experimental subjects to investigate the effects of individual and combined supplementation of BS and RYC on growth performance, meat quality, and antioxidant capacity in broilers. At the same time, the key metabolic pathways and molecular mechanisms of BS and RYC in regulating meat quality were further analyzed by using non-targeted metabolomics techniques, which provided a solid theoretical basis for the combined use of BS and RYC to improve the meat quality of yellow-feathered broilers.

## 2. Materials and Methods

All animal experiments were conducted in accordance with protocols approved by the Animal Welfare and Ethics Committee of Inner Mongolia Agricultural University (approval number NND2024124). The animal feeding experiments were conducted in the broiler integrated production experimental room of the College of Animal Science, Inner Mongolia Agricultural University, Inner Mongolia, China.

### 2.1. Feed Additives

The *Bacillus subtilis* was provided by Beihai Yiqiang Biotechnology Co., Ltd. (Beihai, China) and had a viable count of 1 × 10^11^ CFU/g. The *Rhodotorula* yeast culture was provided by the Institute of Animal Science of CAAS (Beijing, China). The *Rhodotorula* yeast culture (β-glucan ≥ 1.45 mg/g, mannan oligosaccharides ≥ 0.33 mg/g, carotenoids ≥ 1.60 mg/g) was provided by the State Key Laboratory of Animal Nutrition at the Beijing Institute of Animal Husbandry and Veterinary Medicine, CAAS. The RYC was produced by fermenting *Rhodotorula mucilaginosa* in a liquid broth with soybean meal as the solid substrate. The final product contained yeast cell walls, cellular metabolites, and residual medium components [21,22].

### 2.2. Experimental Design

In this study, 192 one-day-old yellow-feathered broilers with similar body weights (40 ± 0.64 g) were randomly divided into four treatment groups, with 6 replicates for each treatment and 8 chicks per replicate (half male and half female). The CON (control) group: basal diet; the BS (*Bacillus subtilis*) group: basic diet + 5 × 10^9^ CFU/kg *Bacillus subtilis*; the RYC (*Rhodotorula* yeast culture) group: basic diet + 5000 mg/kg *Rhodotorula* yeast culture; the MIX (mixture) group: a basic diet + 5 × 10^9^ CFU/kg *Bacillus subtilis* + 5000 mg/kg *rhodotorula* yeast culture. Both BS and RYC were uniformly mixed into the diet in solid form at designed proportions for feeding convenience, following the same mixing method as described by Su et al. [23]. The implementation standard of basic diet was referred to the Feeding Standard of Chicken (NY/T33-2004) [24,25] and the Nutrition Requirement for Yellow-Feather Broilers (NY/T 3645-2020) [26,27]. The formulation and nutritional levels of the basal diet were presented in Table 1. All broilers were raised in a uniformly ventilated poultry house under identical environmental conditions. The ambient temperature was initially maintained at 33 °C during the first week, and was subsequently reduced by 3 °C each week until it stabilized at 21 °C. The experimental period was 56 days, comprising two distinct stages: the initial stage (1–28 d) and the subsequent stage (29–56 d). Throughout the 56-day experimental period, all birds were given diets and water freely.

### 2.3. Sample Collection

The birds were weighed and recorded during the initial stage and the subsequent stage. The body weight (BW) of each chicken was measured before feeding the feed on the morning of days 0, 28, and 56, respectively, to calculate the average daily gain (ADG), average daily feed intake (ADFI), and feed conversion rate (FCR). On the 56th of the experiment, 1 yellow-feathered broiler, whose weight was close to the average, was chosen from each replicate, and subwing vein blood collection. The blood samples were centrifuged for 10 min at 1237× *g* in sealed tubes to prevent potential contamination during centrifugation or sample evaporation. The resulting serum was then aliquoted and stored at −20 °C for subsequent analysis. Following the administration of anesthesia, the selected yellow-feathered broilers were euthanized via carotid artery transection, and then the chest muscle samples were quickly cut from the left side of the carcass and stored at 4 °C for meat quality determination. The remaining portion was preserved at −80 °C for subsequent analysis of antioxidant enzyme activity in the breast muscle.

### 2.4. Meat Quality Measurement

The pH of the broiler breast was measured at 45 min and 24 h after slaughter, and the samples were stored at 4 °C for 24 h. The pH in muscle tissue was directly measured using a portable pH meter (Matthaus, Pöttmes, Germany). Meat color parameters, including redness (a*), yellowness (b*), and lightness (L*), were measured using a portable chromameter (SMY-2000sf, SMY Technology Co., Ltd., Beijing, China). There was a measurement of the shear force conducted using a digital muscle tenderness instrument (C-LM3B, Northeastern University, Shenyang, China), and the drip loss and cooking loss were evaluated following the methodology outlined by Mao et al. [28]. The pressing loss analysis was performed using a computer-based pressure test instrument (RH-1000, Runhu Instrument Co., Ltd., Guangzhou, China).

### 2.5. Antioxidant Capacity

Catalase (CAT) activity, superoxide dismutase (SOD) activity, glutathione peroxidase (GSH-Px) activity, total antioxidant capacity (T-AOC) and malondialdehyde (MDA) content were analyzed according to the instructions of the reagent kits (Nanjing Jiancheng Bioengineering Institute, Nanjing, China). All spectrophotometric measurements were conducted using a Synergy H1 hybrid multi-mode microplate reader (BioTek Instruments, Winooski, VT, USA) equipped with Gen5 software (version 3.11). Each sample was analyzed in triplicate, and enzyme activities were calculated strictly according to the manufacturer’s specified formulas.

### 2.6. Metabolomics Analysis of Serum Samples

We conducted metabolome analysis using ultra-performance liquid chromatography tandem mass spectrometry (UPLC-MS/MS) [29]. There were 100 μL serum samples accurately removed and placed in a 1.5 mL centrifuge tube, to which 400 μL of extract containing 0.02 mg/mL internal standard L-2-(chlorophenylalanine) (methanol: acetonitrile = 1:1) was subsequently added. Then, the vortex was mixed for 30 s and subsequently extracted by low-temperature ultrasound (5 °C, 40 KHz) for 30 min. Next, the sample was placed at −20 °C for 30 min and centrifuged at 13,000× *g* for 15 min at 4 °C. The supernatant was removed and dried with nitrogen, and then 120 μL (methanol: acetonitrile = 1:1) was added for reconstitution. There was vortexing and mixing of the sample for 30 s, followed by extraction using low-temperature ultrasound (5 °C, 40 KHz) for 5 min. Finally, after centrifugation at 13,000× *g* for 15 min at 4 °C, the supernatant was carefully transferred to the sample bottle for LC-MS/MS analysis. In addition, each sample was mixed with 20 μL supernatant as a quality control sample (QC), and then analyzed using the same method as the analysis sample to monitor the stability of the analysis. The LC-MS/MS analysis of the sample was conducted on a Thermo UHPLC-Q Exactive HF-X system equipped with an ACQUITY HSS T3 column (100 mm × 2.1 mm i.d., 1.8 μm; Waters, Milford, MA, USA) at Majorbio Bio-Pharm Technology Co. Ltd. (Shanghai, China). The chromatographic conditions were as follows: 3 μL samples were separated on an HSS T3 column (100 mm × 2.1 mm i.d., 1.8 µm) prior to mass spectrometric detection. The mobile phase system consisted of A 95% water + 5% acetonitrile (containing 0.1% formic acid) and B 47.5% acetonitrile + 47.5% isopropanol + 5% water (containing 0.1% formic acid). The analysis was performed at a flow rate of 0.40 mL/min with the column temperature maintained at 40 °C.

### 2.7. Date Analysis

All datasets were initially assessed for normality and homogeneity of variance using Shapiro-Wilk’s test and Levene’s test, respectively, with *p* > 0.05 indicating normally distributed data [30]. Subsequently, one-way analysis of variance (ANOVA) was performed to compare the four experimental groups [31]. Following ANOVA, post-hoc comparisons were conducted using Tukey-Kramer’s multiple range test to identify specific differences among treatment groups. Statistical analyses of growth performance, breast meat quality, and antioxidant parameters in yellow-feathered broilers were performed using IBM SPSS Statistics 26.0 (SPSS Inc., Chicago, IL, USA). The table data are presented as the mean and standard error of mean (SEM). *p* < 0.05 was defined as a significant difference, and 0.05 ≤ *p* < 0.10 was defined as a trend of difference.

For the metabonomic data, the raw data were imported into the metabolic group processing software Progenesis QI v3.0 (Waters Corporation, Milford, CT, USA) for pretreatment. Secondly, there was the application of the R package “ropls” (Version 1.6.2) to conduct Partial Least Squares Discriminant Analysis (PLS-DA) on the preprocessed data matrix. The selection of significantly different metabolites was based on the variable importance (VIP) obtained by the PLS-DA model, and the student’s test *p*-value to determine the VIP > 1, *p* < 0.05 metabolites were significantly different metabolites.

## 3. Results

### 3.1. Growth Performance

As shown in Table 2, in the initial phase of the trial (days 1–28), compared with the CON group, the BW and ADG of broilers in the BS group were markedly increased (*p* < 0.05). Moreover, the BW and ADG of broilers in the RYC and MIX groups at 28 days of age exhibited a tendency to rise. For both the initial and subsequent stages, there were no significant differences in ADFI and FCR between the BS, RYC, and MIX groups and the CON group (*p* > 0.05).

### 3.2. Meat Quality

As shown in Table 3, compared with the CON group, the shear force and water loss rate of the MIX group were significantly decreased (*p* < 0.05). Although the shear force and water loss rate of the BS and RYC groups showed a downward trend, the differences were not statistically significant. Similarly, the a* of breast meat color in the MIX group had an increasing tendency compared with the CON group (*p* = 0.059). The pH, cooking loss, and drip loss of the BS, RYC, and MIX groups showed no significant differences compared with the control group (*p* > 0.05).

### 3.3. Antioxidant Capacity of Breast Muscle

As shown in Table 4, compared with the CON group, the BS group significantly enhanced the activities of CAT and SOD on day 28 (*p* < 0.05); the RYC and MIX groups steeply increased CAT activity on day 28 (*p* < 0.05). In the subsequent stage, compared with the CON group, the BS group markedly increased GSH-Px activity, and the RYC group showed a tendency towards increased GSH-Px activity (*p* < 0.05). The MIX group exhibited a trend towards enhanced T-AOC activity (*p* = 0.067).

### 3.4. Serum Metabolome

**Figure 1:**
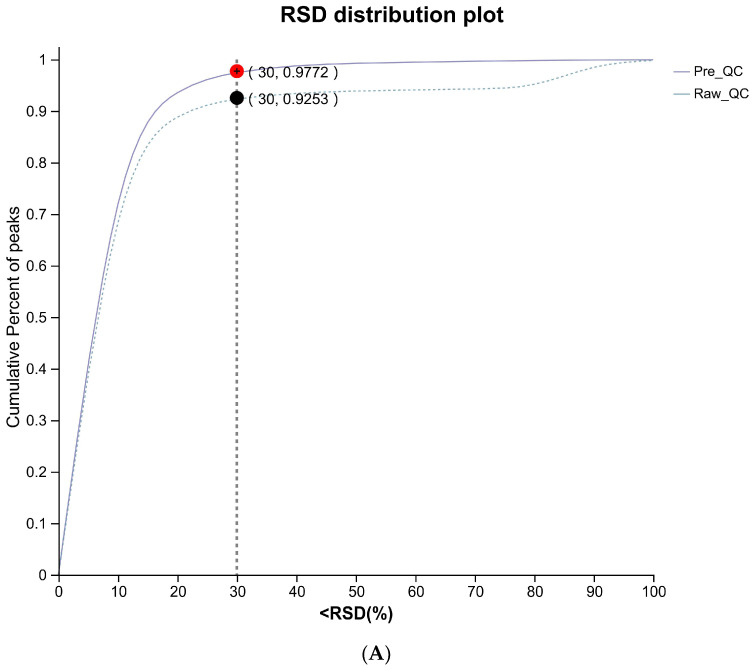

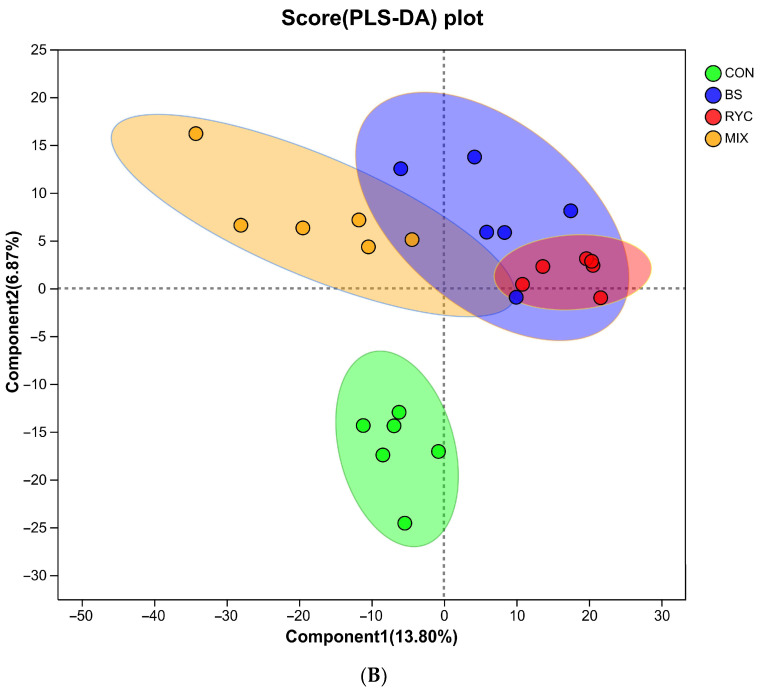
(**A**) RSD distribution of quality control samples for evaluating method repeatability. (**B**) PLS-DA of serum metabolites from broilers (*n* = 6) in CON, BS, RYC, and MIX groups.

The system stability during the entire sample batch process was verified by the QC samples. As shown in Figure 1A, the relative standard deviation (RSD) value of >70% QC samples was <30%, indicating that the proposed method has good stability. Then, Partial Least Squares Discriminant Analysis (PLS-DA) was performed to further explore the subtle differences in metabolic profiles between groups in Figure 1B. There is a supervised discriminant analysis method known as PLS-DA, which is a type of multivariate statistical analysis method. The above statistical analysis methods could comprehensively reflect the overall differences between groups and the degree of variation within the group, so as to comprehensively evaluate the distribution characteristics and discrete trends of the sample set [32]. In this study, the PLS-DA model showed a significant separation between the three experimental groups and the CON group, indicating that the serum metabolic patterns of the BS group, the RYC group, and the MIX group were significantly different from the CON group.

**Figure 2:**
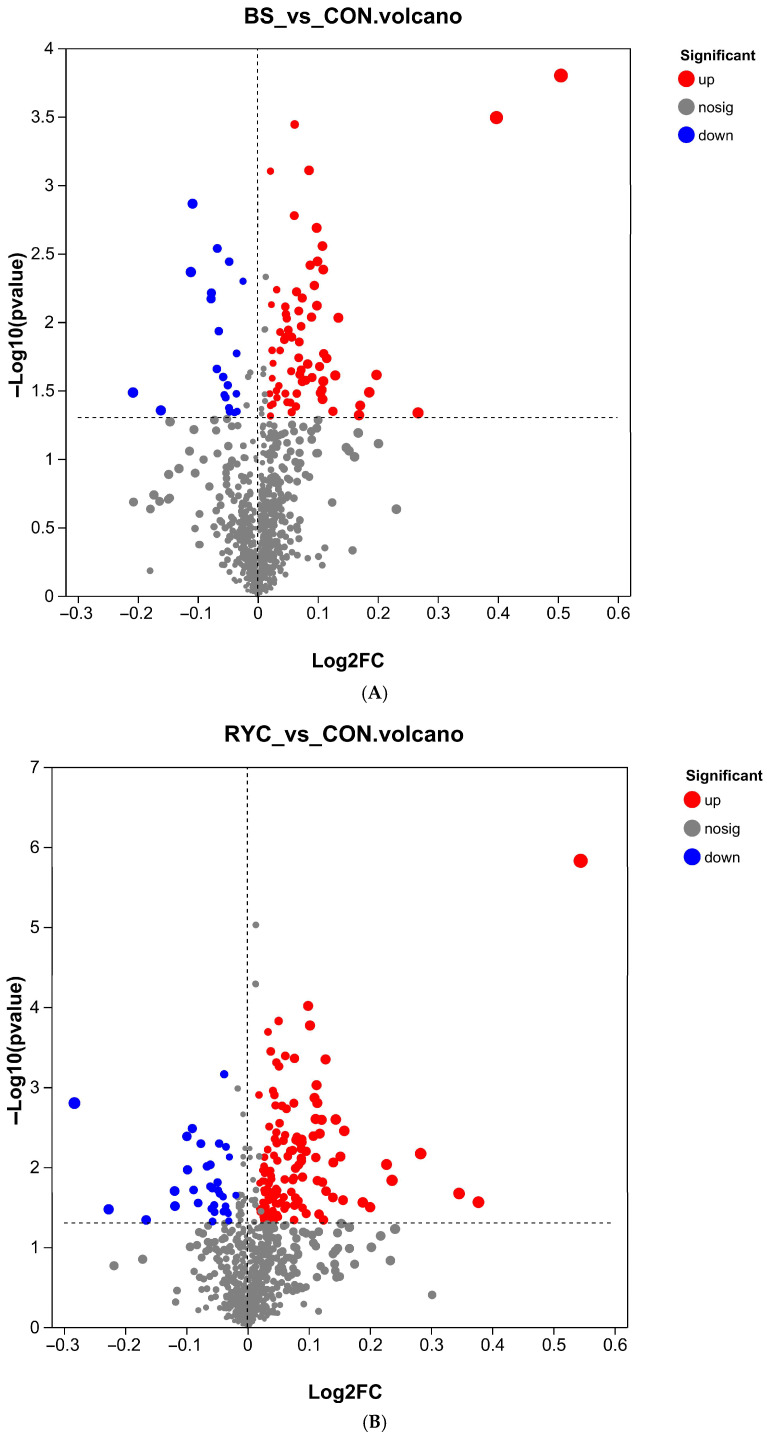

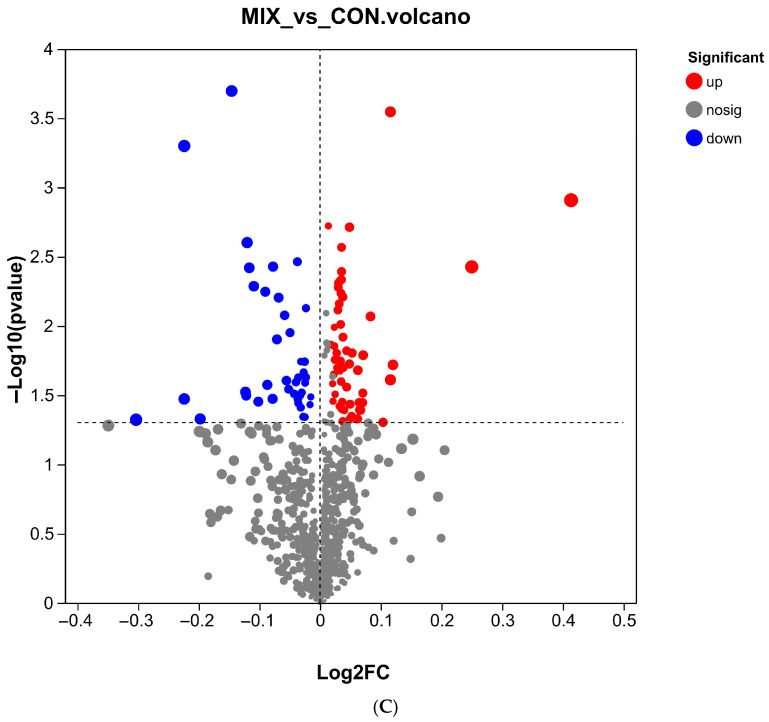
(**A**) Volcano plots of the same biomarkers in BS group and CON group. (**B**) Volcano plots of the same biomarkers in RYC group and CON group. (**C**) Volcano plots of the same biomarkers in MIX group and CON group.

The results of metabolomics analysis showed that compared with the CON group, 829, 1308, and 841 differential metabolites were identified in the BS group, RYC group, and MIX group, respectively, of which 90, 169, and 96 metabolites were successfully annotated. Further analysis showed that 69 metabolites were significantly up-regulated and 21 metabolites were significantly down-regulated in the BS group (Figure 2A). In the RYC group, 136 were up-regulated and 33 were down-regulated (Figure 2B); the MIX group showed 56 up-regulated and 40 down-regulated metabolites (Figure 2C).

**Figure 3:**
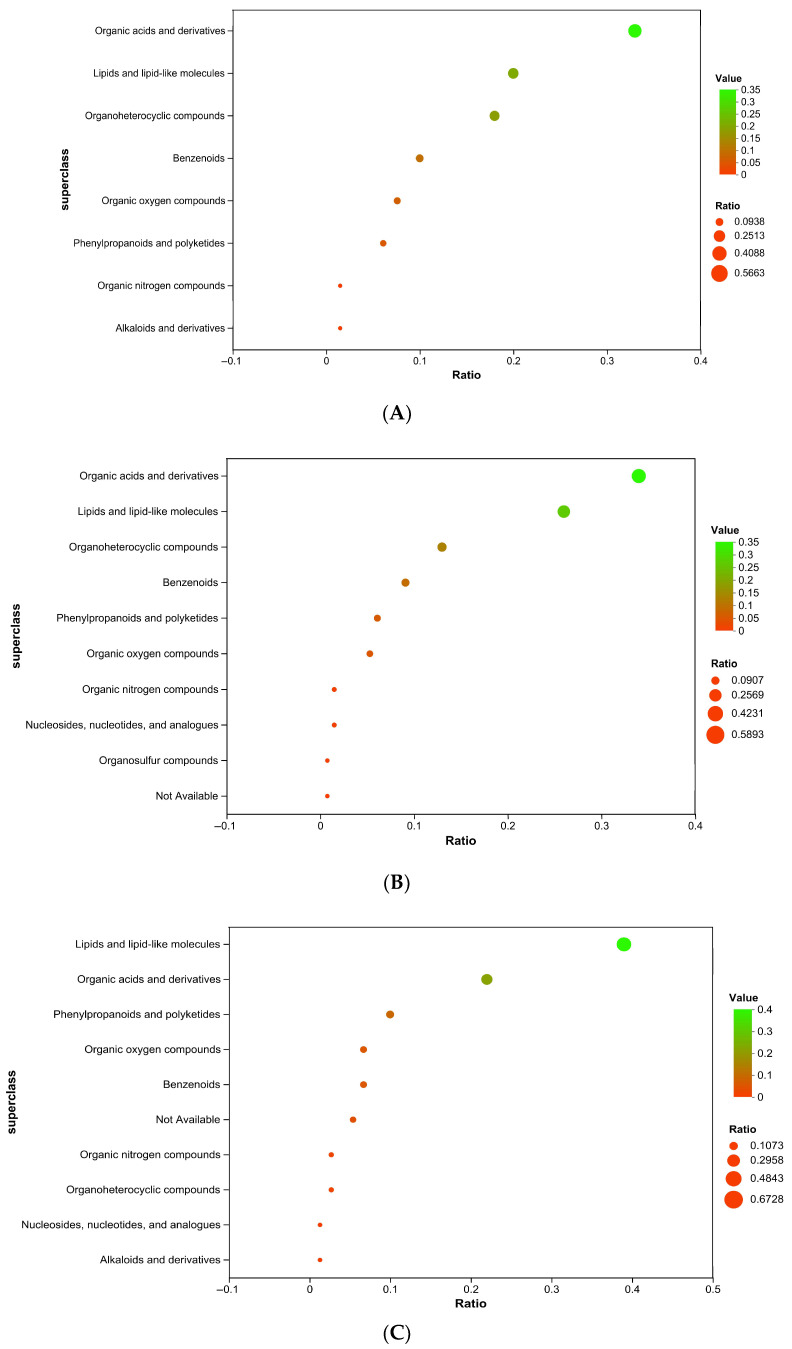
(**A**) The relative abundance of serum metabolites of broilers in BS group and CON group. (**B**) The relative abundance of serum metabolites of broilers in RYC group and CON group. (**C**) The relative abundance of serum metabolites of broilers in MIX group and CON group.

The results of metabolomics analysis based on the HMDB database showed that the high-abundance metabolites in each experimental group showed significant differences in the distribution of chemical categories. Compared with the CON group, 65 high-abundance metabolites were identified in the BS group (Figure 3A), including 13 organic acids and derivatives, 13 lipids and lipid-like molecules, and 12 organoheterocyclic compounds. In the RYC group, 131 high-abundance metabolites were detected (Figure 3B), of which organic acids and derivatives and lipids and lipid-like molecules were dominant. The 74 high-abundance metabolites in the MIX group were mainly lipids and lipid-like molecules, and organic acids and derivatives (Figure 3C). The characteristic metabolite categories shared by the three groups included benzenoids, phenylpropanoids, and polyketides, and organic oxygen compounds, while alkaloids and derivatives, nucleosides, nucleotide, and analogues were only detected in specific groups.

**Figure 4:**
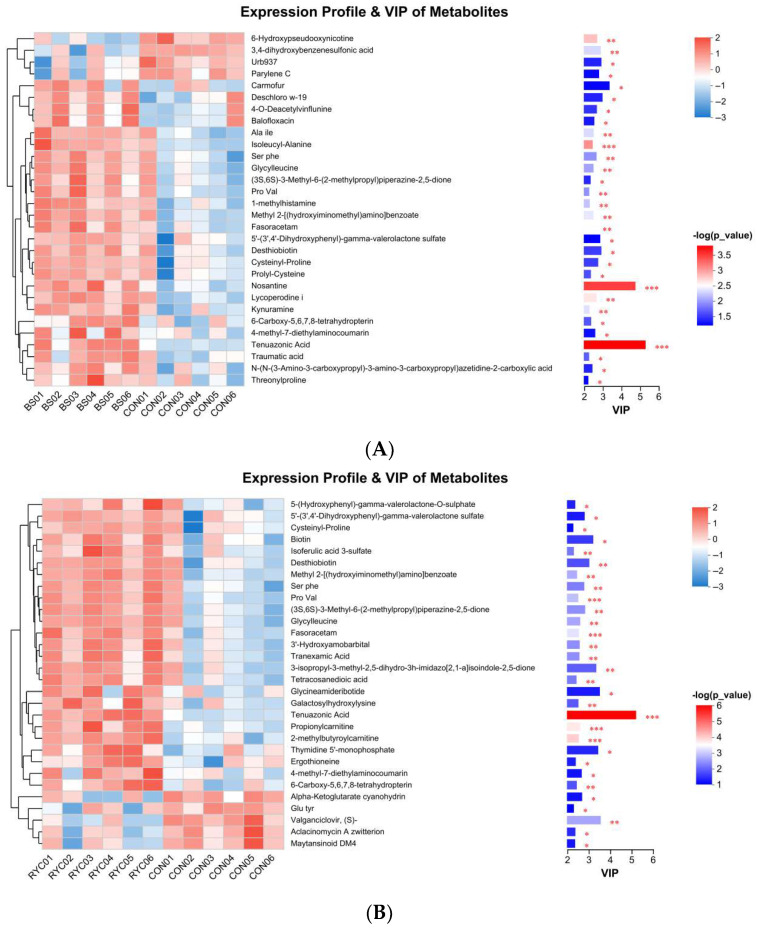

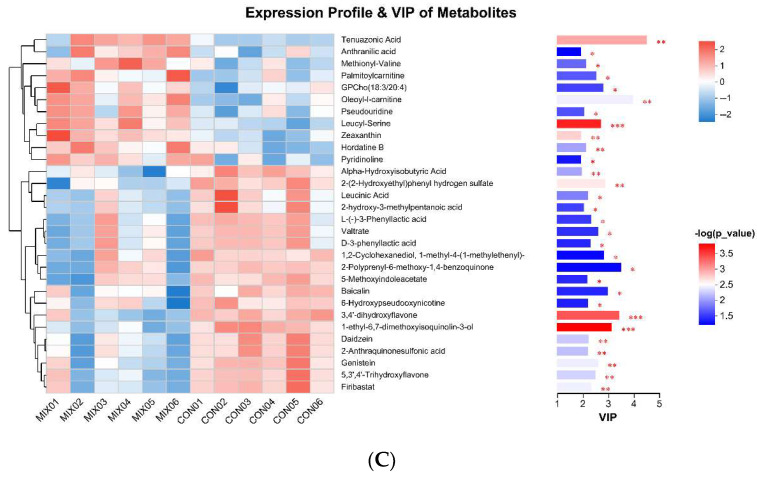
(**A**) Hierarchical cluster analysis and heat map analysis were performed on the identified 30 metabolites, and significant differences were found between serum samples (*n* = 6) from broilers of the BS group and the CON group. (**B**) Hierarchical cluster analysis and heat map analysis were performed on the identified 30 metabolites, and significant differences were found between serum samples (*n* = 6) from broilers of the RYC group and the CON group. (**C**) Hierarchical cluster analysis and heat map analysis were performed on the identified 30 metabolites, and significant differences were found between serum samples (*n* = 6) from broilers of the MIX group and the CON group. Each row represents a metabolite, and the columns are individual samples for two groups. Levels of significance were defined as * *p* < 0.05, ** *p* < 0.01, and *** *p* < 0.001.

In order to further analyze the distribution characteristics of differential metabolites with biological activity, the chemical composition heat map analysis of each experimental group and CON group was carried out in this study. Metabolites with VIP > 1 were considered to be important. The results showed that 26 compounds in the serum of broilers in the BS group were significantly increased, and 4 compounds were decreased (Figure 4A). The RYC group showed 25 increased and 5 decreased metabolites (Figure 4B); in the MIX group, 11 compounds increased but 19 decreased significantly (Figure 4C). This differential metabolite change trend suggests that different treatments have a specific regulatory effect on the serum metabolic profile of broilers.

**Figure 5:**
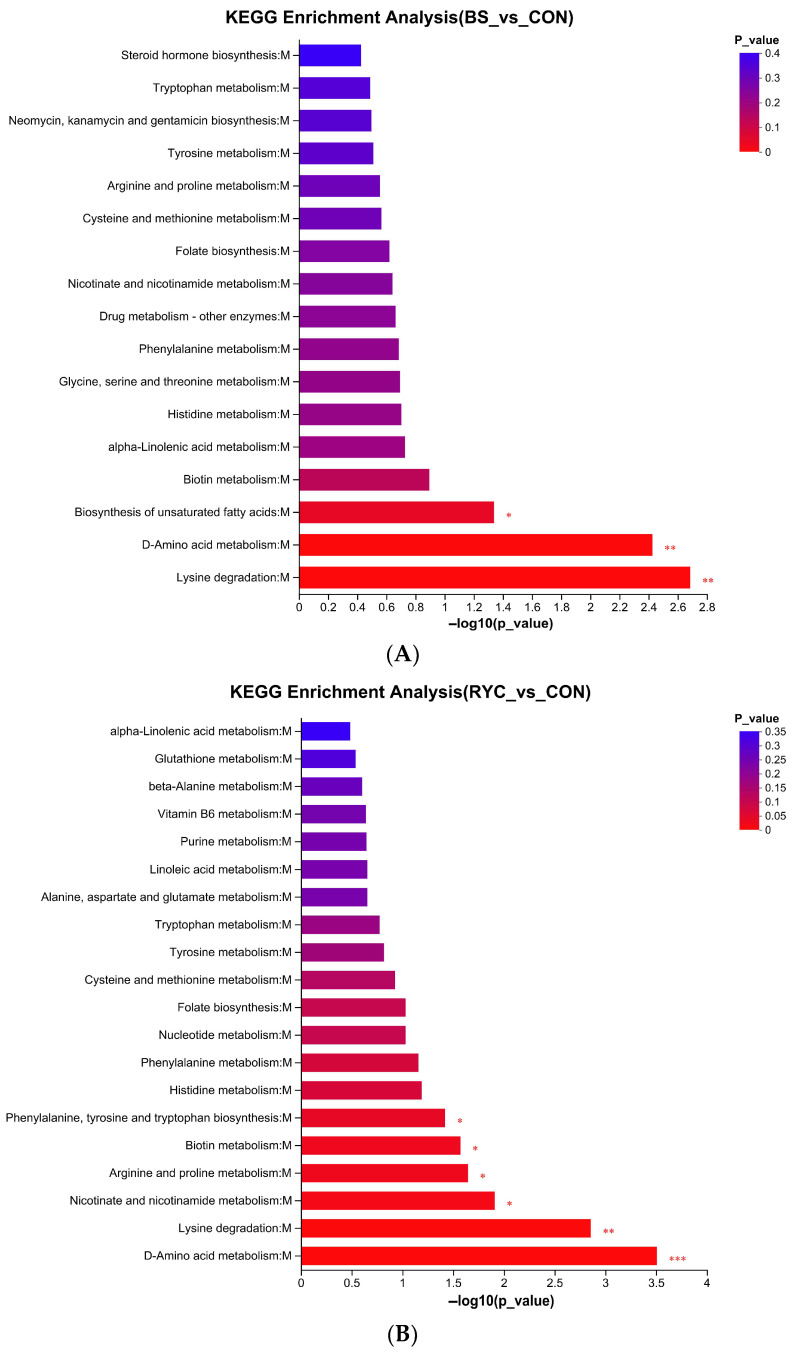

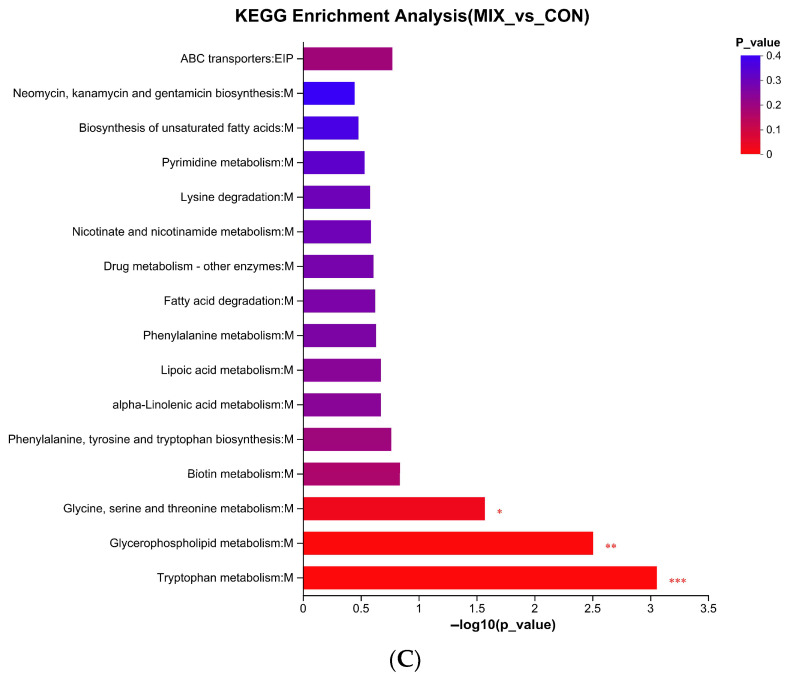
(**A**) Compared with the CON group, the KEGG pathway enrichment data in the BS group identified three significant pathways. (**B**) Compared with the CON group, the KEGG pathway enrichment data in the RYC group identified six significant pathways. (**C**) Compared with the CON group, the KEGG pathway enrichment data in the MIX group identified three significant pathways. Generally, the *p* < 0.05 was considered to be a significant enrichment term; the ordinate is the KEGG pathway. Levels of significance were defined as * *p* < 0.05, ** *p* < 0.01, and *** *p* < 0.001.

Based on KEGG pathway analysis, this study identified significant metabolic pathways in each group compared with the CON group (*p* < 0.01). The results showed that the differential metabolites in the BS group and the RYC group were mainly enriched in lysine degradation and D-Amino acid metabolism pathway (Figure 5A,B), while the MIX group was significantly enriched in tryptophan metabolism and glycerophospholipid metabolism pathway (Figure 5C). These results indicate that different treatment groups exert their biological effects by regulating specific metabolic pathways.

### 3.5. The Relationship Between the Metabolites and Meat Quality

**Figure 6:**
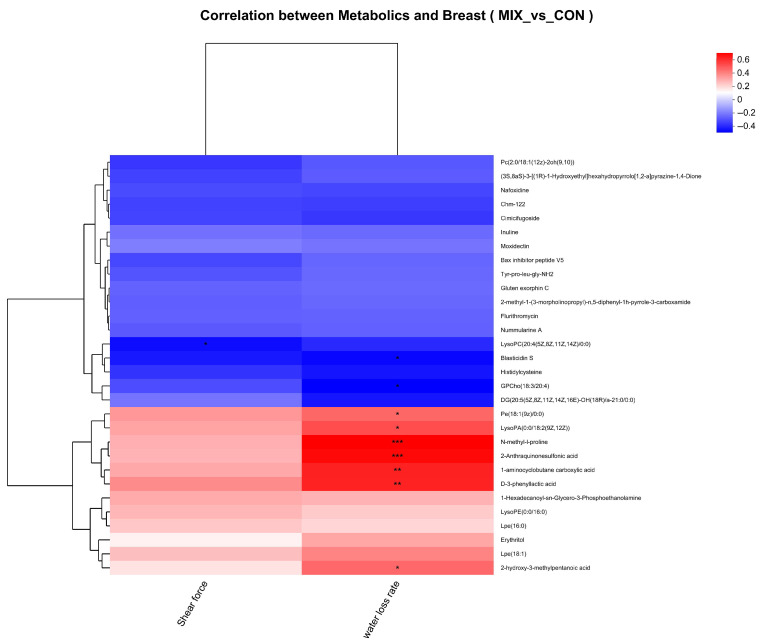
The correlations between metabolites and meat quality in the breast muscle of broilers were compared between MIX group and CON group. Levels of significance are defined as * *p* < 0.05 and ** *p* < 0.01, and *** *p* < 0.001.

Figure 6 shows the correlation between metabolites and meat quality in the breast muscle of broilers in the MIX group and the CON group. LysoPC(20:4(5Z,8Z,11Z,14Z)/0:0) negatively correlated with the shear force (*p* < 0.05). GPCho(18:3/20:4) and Blasticidin S negatively and 1-aminocyclobutane carboxylic acid, 2-hydroxy-3-methylpentanoic acid, N-methyl-l-proline, D-3-phenyllactic acid, 2-Anthraquinonesulfonic acid, Pe(18:1(9z)/0:0) as well as LysoPA(0:0/18:2(9Z,12Z)) positively associated with the water loss rate (*p* < 0.05).

## 4. Discussion

Probiotics are living microorganisms included in animal diets as feed additives or supplements. They were often referred to as direct-feeding microorganisms and were beneficial to both the host and the bacteria [33]. In the present study, it was observed that there were no substantial differences in feed intake among the CON, BS, RYC, and MIX groups. This finding indicates that the feed intake of broilers in each group was maintained at a relatively consistent level during the trial period, thereby ruling out the possibility that the changes in amino acid levels were due to differences in feed intake. Instead, the trial results were closely related to the effects of the feed additives administered. The result of this study demonstrated that the BS-supplemented diets significantly enhanced the BW and ADG at the initial stage. There was an increasing trend observed in both BW and ADG for broilers in the RYC and MIX groups. These findings demonstrate that BS supplementation can effectively improve the growth performance of yellow-feathered broilers, which is consistent with previous research findings. Specifically, Wang et al. [34] reported that dietary supplementation with *Bacillus subtilis* K3 strain at 1 × 10^8^–1 × 10^9^ CFU/kg significantly increased the BW of AA broilers at 6 weeks of age. Similarly, Zhang et al. [35] demonstrated that supplementation with *B. subtilis* in the raw product containing between 2 × 10^10^ and 3 × 10^10^ CFU/g significantly enhanced the final body weight and ADG of 42-day-old AA broilers. During the later growth stages, BS and RYC primarily exert their effects by enhancing intestinal barrier integrity and activating immune responses rather than promoting nutrient utilization [23]. This physiological process may lead to a moderate reduction in protein deposition efficiency due to energy repartitioning [36]. These results suggest the potential need for dynamic adjustment of BS and RYC supplementation levels according to different growth phases to better meet the nutritional requirements of yellow-feathered broilers.

Consumer perception of meat products, particularly freshness, color, texture, and other sensory attributes, significantly influences purchasing decisions and quality judgments [37]. Protein functionality in meat is pH-dependent, directly affecting water-holding capacity. After slaughter, the muscle tissue produced lactic acid due to glycolysis, resulting in a decrease in pH value. When the pH is close to the isoelectric point of myosin (pI = 5.4), the net charge of the protein is zero due to the balance of positive and negative charge groups, and the positive and negative charges in the molecule attract each other. At the same time, the tightness of the muscle structure caused the water inside the muscle fiber to be squeezed out, which greatly reduced the water retention ability [38]. The change of water holding capacity, drip loss, and cooking loss of meat were usually closely related to the change of pH of meat [39]. It was found by Wang et al. [40] that meat quality was enhanced by BS through improving meat color, water holding capacity, and reducing shear force. However, the study by Fang et al. [41] demonstrated that dietary supplementation with 0.015%, 0.030%, and 0.045% *Bacillus subtilis* ACCC 11025 in the basal diet did not exert significant effects on breast meat quality of AA broilers. Similarly, Shi [42] reported that neither the addition of 2.5% and 5.0% novel Saccharomyces cerevisiae culture substrate nor 2.5% and 5.0% novel S. cerevisiae culture in corn-soybean meal basal diets significantly influenced breast muscle pH value or drip loss in Cherry Valley ducks. These findings are consistent with the results obtained in our current study. Furthermore, Mohammed et al. [43] observed that supplementation with 0.25 g/kg and 0.50 g/kg *B. subtilis* PB6 in conventional feed significantly decreased thigh muscle pH while improving water-holding capacity in Ross 708 broilers. Based on these collective findings, we speculate that the absence of significant alterations in breast muscle pH may primarily account for the lack of significant differences in water-holding capacity, cooking loss, and drip loss observed in the meat products.

In addition to the above factors, meat color and shear force were also critical factors for measuring meat quality. The present study demonstrated that although the BS and RYC groups showed a tendency to reduce breast muscle shear force and water loss rate compared with the CON group, the differences did not reach statistical significance. These findings are consistent with previous research conclusions. Chen et al. [20] reported that daily supplementation with 10, 20, and 40 g/day RYC before morning feeding significantly decreased the shear force of the longissimus dorsi muscle in sheep. Similarly, Mohammed et al. [43] found that dietary supplementation with different doses of *B. subtilis* PB6 in Ross 708 broilers effectively improved thigh muscle shear force. When BS and RYC were mixed, the shear force and water loss rate of breast muscle could be significantly reduced. Notably, this study observed an increasing trend in the a* of the RYC and MIX groups, although statistical significance was not achieved. Consistent with these findings, prior research has indicated that β-carotene has the potential to elevate the a* in beef, a finding that aligns with the results of the present experiment [44].

Reactive oxygen species (ROS) are chemically active oxygen-containing molecules with many beneficial functions in living organisms and serve as a key factor in cell signaling and metabolism. However, excessive ROS could lead to oxidative damage, affect poultry meat quality (such as white stripes, wood breast), intestinal health, and reduce production performance and meat quality [45]. The enzymatic antioxidant system (including CAT, SOD, GSH-Px, etc.) converts ROS into harmless substances such as water and oxygen. This system eliminates excess reactive oxygen free radicals generated in the body, prevents oxidative damage caused by free radical accumulation, maintains redox balance, and ensures organismal health [46]. The present study revealed significant variations in antioxidant enzyme activities of yellow-feathered broilers across different growth stages, reflecting the dynamic regulatory characteristics of the antioxidant defense system. During the early developmental phase, the broilers undergo a critical period of environmental acclimatization characterized by incomplete maturation of major organ systems. At 28 days of age, the broilers were presumably undergoing a transitional adaptation phase in response to the feed alteration, which induced moderate oxidative stress. In response, the birds upregulated CAT and SOD activities to establish a more robust antioxidant defense system, a finding consistent with previous reports by Wang et al. [40] in poultry. Xiong et al. [47] further demonstrated that dietary supplementation with 0.5%, 1%, 2%, and 4% Saccharomyces cerevisiae culture significantly enhanced CAT activity in the breast muscle of Sichuan white geese. The stable GSH-Px levels during this phase suggested that the antioxidant defense mechanisms had successfully contained further oxidative stress progression in the early stage. By 56 days of age, the broilers had adapted to dietary and environmental conditions, leading to stabilized oxidative stress levels, which may explain the non-significant changes in CAT and SOD activities. Notably, the BS group showed significantly elevated GSH-Px activity, while the RYC group exhibited an increasing trend, and the MIX group demonstrated a tendency for enhanced T-AOC capacity. These observations indicate that combined BS and RYC supplementation may provide more comprehensive antioxidant effects during prolonged feeding periods. The compensatory mechanisms and overall antioxidant capacity improvement reflect the dynamic regulatory capacity of the antioxidant defense system during long-term adaptation, revealing stage-specific metabolic and antioxidant requirements in yellow-feathered broilers. It is noteworthy that meat quality characteristics are closely associated with muscle oxidative status [48,49,50], particularly as myoglobin oxidation influences meat color changes [51]. In our study, the MIX group at 56 days showed a tendency for higher T-AOC compared to the CON group, which may explain the observed trend of increased a* in meat color, thereby corroborating our earlier research findings [20].

It was an innovative combination strategy to add BS and RYC as probiotics to the feed, aimed at exploring the potential effects of the combination of BS and RYC on the meat quality of yellow-feathered broilers. This research further explored the changes in metabolites and metabolic pathways of meat quality in broilers, dietary supplementation with BS and RYC in the diet. The effect of probiotic dietary supplementation on meat quality has rarely been investigated, as notably shown by previous studies. At the same time, its role in serum metabolomics had rarely been explored [40,41]. In recent years, metabolomics has become a prominent research hotspot. However, relative to other domains, the utilization of metabolomics in meat quality research remains in its nascent stages [52]. In this study, we observed that the incorporation of BS and RYC, either individually or in combination, into the feed led to enhancements in broiler growth performance and meat quality. Furthermore, we performed metabolomics analysis on serum samples from the three experimental groups and the CON group to identify differential metabolites and their enrichment pathways. Through the evaluation of RSD and PLS-DA models, the metabolomics map was demonstrated to possess good stability and reliability.

The accumulation of lipid and lipid-like molecules, organic acids and derivatives, and organoheterocyclic compounds was larger in both BS and RYC groups. The accumulation of benzenoids, organic oxygen compounds, phenylpropanoids and polyketides, alkaloids and derivatives, organic nitrogen compounds, and nucleosides, nucleotides, and analogues was smaller. This aligned with the results reported by Tang et al. [53] and An et al. [54]. In the MIX group, the accumulation of lipid and lipid-like molecules and organic acids and derivatives was larger, while the accumulation of other compounds was smaller. Subsequently, there were heatmaps generated for the three experimental groups relative to the CON group to visually depict the distribution of critical differential metabolites with biological activity in each category. The results showed that the differences between the BS group and the CON group, as well as between the RYC group and the CON group, were mainly reflected in the abundance of organic acids and derivatives. In contrast, the differences between the MIX group and the CON group were primarily manifested in the abundance of lipids and lipid-like molecules, including GPCho(18:3/20:4), Palmitoylcarnitine, Zeaxanthin, 2-Polyprenyl-6-methoxy-1,4-benzoquinone, 1,2-Cyclohexanediol, 1-methyl-4-(1-methylethenyl)-, 2-hydroxy-3-methylpentanoic acid, Valtrate, and Leucinic Acid. Specifically, GPCho(18:3/20:4), Palmitoylcarnitine, and Zeaxanthin increased, while the remaining five metabolites decreased.

GPCho(18:3/20:4) was a phospholipid molecule that belongs to the phosphatidylcholine (PC) class. The hydrolysis of phospholipid molecules in fresh meat after slaughter disrupted the structural integrity of mitochondrial and cellular membranes. This process facilitates Ca^2+^ release into the cytosol, thereby accelerating the activation of endogenous proteolytic enzymes and the degradation of myofibrillar proteins and promoting meat tenderization [55]. Palmitoylcarnitine was an intermediate product of fatty acid metabolism, and its level was closely related to the PPAR signaling pathway. PPAR was a kind of nuclear receptor that regulates fatty acid oxidation, lipid metabolism, and energy balance. Research has confirmed that the activation of the PPAR signaling pathway promoted fatty acid oxidation and increased intramuscular fat content, thereby improving meat tenderness [56]. In this study, the 56 d MIX group showed significantly higher total antioxidant capacity than the other experimental groups, and its shear force was significantly reduced, indicating significant improvement in meat tenderness. This phenomenon may be closely related to the biological characteristics of zeaxanthin. As an important member of the carotenoid family, zeaxanthin has significant antioxidant activity [57]. The conjugated double bond system in its molecular structure could effectively quench singlet oxygen and scavenge free radicals [58]. Therefore, it is plausible that zeaxanthin enhances meat quality through the augmentation of antioxidant defense mechanisms.

Further enrichment analysis showed that dietary supplementation with BS or RYC alone significantly affected the two pathways of D-amino acid metabolism and lysine degradation. L-amino acids interacted with salt-soluble proteins to regulate intermolecular forces, thereby enhancing protein solubility, preventing excessive aggregation, and upgrading the quality of gel-type meat products [59]. Previous studies have demonstrated that amino acid racemase secreted by BS converts L-amino acids into D-amino acids [60]. In our study, it was speculated that D-amino acids may be derived from the conversion of L-amino acids, which could increase the diversity of amino acids. It may affect the antioxidant pathway and protein modification, and was ultimately critical for the regulation of breast muscle quality of broilers. The decrease of lysine level may result in ROS accumulation, which may lead to muscle oxidative damage [61]. However, in this experiment, the individual supplementation of BS or RYC showed limited effects on meat quality improvement. This may be attributed to counteracting biological effects between the D-amino acid metabolism and lysine degradation pathways. In contrast, the combined addition of BS and RYC significantly affected the two metabolic pathways of glycerophospholipid metabolism and tryptophan metabolism. Studies have found that glycerophospholipid is an important part of intramuscular fat, and its metabolism directly affects the content and composition of intramuscular fat. By regulating glycerophospholipid metabolism, the deposition of intramuscular fat can be increased, thereby improving meat tenderization and flavor development [62,63]. At the same time, tryptophan and its metabolites were crucial for regulating muscle growth and development [64]. Changes in tryptophan metabolism may indirectly influence animal growth performance and meat quality by regulating related neurotransmitters and hormone levels [65]. Notably, the MIX group demonstrated comprehensive improvements, including enhanced meat color, tenderness, and water-holding capacity in yellow-feathered broilers. More importantly, through the coordinated regulation of glycerophospholipid and tryptophan metabolism, this treatment significantly upgraded the overall meat quality and market value.

In addition, the results of correlation analysis based on PLS-DA in this study showed that in the breast muscle, we found that PC metabolites were negatively correlated with shear force and water loss compared to the CON group. These metabolites exhibited a negative correlation with water-holding capacity, indicating that the increase of metabolites may be related to lower cooking loss and drip loss, thereby indirectly enhancing the water-holding capacity. There had been widespread recognition of the importance of glycerophospholipid metabolism and its related lipid metabolites [66,67]. Among them, PC metabolites were enriched and identified, garnering considerable attention due to their significant impact on meat quality [68]. Although this study initially established a digital relationship between metabolites and meat quality, the underlying biological mechanisms still need to be further explored. Furthermore, these observed associations may be subject to genetic regulation, necessitating future resolution through integrated multi-omics approaches combining transcriptomics and metabolomics.

## 5. Conclusions

The results showed that both BS and RYC could effectively improve the meat quality of yellow-feathered broilers, and there was a superior effect when the two were applied together compared to their individual use. Metabolomics analysis showed that BS and RYC combined affected related serum metabolites by regulating glycerophospholipid metabolism and tryptophan metabolism pathways. In addition, the combined application of BS and RYC also significantly improved the antioxidant level in broilers, enhanced the water holding capacity, and tenderness of broilers. These improvements not only improved the meat quality of yellow-feathered broilers but also extended their shelf life. In conclusion, the simultaneous addition of BS and RYC could better enhance the meat quality of broilers and enhance the antioxidant level in poultry meat, which provides more possibilities for the development of composite feed additives.

## Figures and Tables

**Table 1:** Composition and nutrient levels of the basal diet (%, as food).

Items	1 to 28 Days of Age	29 to 56 Days of Age
Ingredients		
Corn	58.95	60.90
Soybean oil	3.00	3.00
Soybean meal	23.00	20.00
Cottonseed cake	6.00	6.00
Rapeseed cake	5.00	6.00
Limestone	1.07	1.22
CaHPO_4_	1.90	1.80
NaCl	0.37	0.37
Choline	0.11	0.11
Vitamin premix ^1^	0.10	0.10
Mineral premix ^2^	0.50	0.50
Total	100.00	100.00
Nutrient levels ^2^ % DM		
ME ^3^ (MJ/kg)	12.26	12.64
Crude protein	21.00	20.00
Crude fiber	7.00	6.00
Calcium	1.05	0.95
Total phosphorus	0.52	0.47
Methionine + Cystine	0.82	0.71

Abbreviations: ^1^ The Vitamin premix provides the following per kg of the diet: VA—9000 IU; VB_1_—3.00 mg, VB_2_—8.00 mg, VB_6_—4.40 mg, VB_12_—0.012 mg, VD_3_—3000 IU, VE—26 IU, VK_3_—1.20 mg, calcium pantothenate—1 mg, nicotinic acid 45 mg. ^2^ The mineral premix provides the following elements per kg of the diet: Fe—100 mg, Cu—10 mg, Mn—120 mg, Zn—108 mg, I—1.50 mg, Se—0.35 mg. ME is calculated; the rest are measured. ^3^ ME (Metabolic energy) was calculated based on data from the Chinese Raw Material Database on animal nutrition.

**Table 2:** Effects of BS and RYC on growth performance of yellow-feathered broilers.

Items	Groups				SEM	*p*-Value
	CON	BS	RYC	MIX		
BW, g						
d1	40.21	40.24	40.04	39.89	0.084	0.438
d28	851.01 ^b^	911.17 ^a^	869.71 ^ab^	890.80 ^ab^	7.295	0.022
d56	2122.14	2046.10	2055.90	2098.10	25.692	0.696
1–28 days						
ADG, g/day	29.15 ^b^	31.15 ^a^	29.63 ^ab^	30.69 ^ab^	0.254	0.021
ADFI, g/day	58.47	59.60	57.89	59.12	3.088	0.998
FCR	2.03	1.97	1.97	1.97	0.018	0.620
29–56 d						
ADG, g/day	45.13	41.11	42.28	43.31	0.936	0.476
ADFI, g/day	133.64	130.51	131.71	131.11	1.725	0.929
FCR	3.26	3.41	3.42	3.32	0.088	0.910
1–56 days						
ADG, g/day	36.65	35.55	35.43	36.49	0.572	0.826
ADFI, g/day	96.05	95.05	94.80	95.12	3.014	0.999
FCR	2.60	2.63	2.64	2.61	0.055	0.995

Abbreviations—CON (control group): basal diet; BS (*Bacillus subtilis* group): basic diet + 5 × 10^9^ CFU/kg *Bacillus subtilis*; RYC (*Rhodotorula* yeast culture group): basic diet + 5000 mg/kg *Rhodotorula* yeast culture; MIX (mixture group): a basic diet + 5 × 10^9^ CFU/kg *Bacillus subtilis* + 5000 mg/kg *Rhodotorula* yeast culture; ^a,b^ Means within a row with different superscripts differ significantly (*p* < 0.05).

**Table 3:** Effects of BS and RYC on meat quality of yellow-feathered broilers.

Items	Groups				SEM	*p*-Value
	CON	BS	RYC	MIX		
pH						
45 min	5.81	6.04	6.16	5.97	0.111	0.742
24 h	5.62	5.69	5.67	5.68	0.028	0.869
Meat color						
L* (lightness)	52.84	55.69	51.96	52.73	0.838	0.438
a* (redness)	2.73	2.69	3.26	3.41	0.120	0.059
b*(yellowness)	13.54	14.90	13.64	15.41	0.499	0.481
Cooking loss, %	18.88	17.95	18.60	17.58	1.137	0.980
Shear force, N	44.69 ^a^	39.56 ^ab^	39.87 ^ab^	32.21 ^b^	1.352	0.020
Drip loss, %	5.31	4.82	4.43	5.03	0.278	0.744
Water loss rate, %	21.00 ^a^	18.17 ^ab^	19.67 ^ab^	15.23 ^b^	0.727	0.021

Abbreviations—CON (control group): basal diet; BS (*Bacillus subtilis* group): basic diet + 5 × 10^9^ CFU/kg *Bacillus subtilis*; RYC (*Rhodotorula* yeast culture group): basic diet + 5000 mg/kg *Rhodotorula* yeast culture; MIX (mixture group): a basic diet + 5 × 10^9^ CFU/kg *Bacillus subtilis* + 5000 mg/kg *Rhodotorula* yeast culture; ^a,b^ Means within a row with different superscripts differ significantly (*p* < 0.05).

**Table 4:** Effects of BS and RYC on antioxidant capacity of breast muscle in yellow-feathered broilers.

Items	Groups				SEM	*p*-Value
	CON	BS	RYC	MIX		
d28						
CAT (U/mg prot)	0.40 ^b^	1.42 ^a^	0.99 ^a^	1.21 ^a^	0.108	0.001
SOD (U/mg prot)	7.90 ^b^	11.61 ^a^	8.98 ^b^	7.63 ^b^	0.412	<0.001
GSH-Px (U/mg prot)	0.94	1.16	1.48	1.08	0.120	0.430
T-AOC (mmol/mg prot)	46.02	50.21	52.20	56.35	1.850	0.269
MDA (nmol/mg prot)	0.15	0.16	0.13	0.15	0.014	0.960
d56						
CAT (U/mg prot)	0.82	0.88	0.78	0.65	0.067	0.720
SOD (U/mg prot)	7.90	7.12	7.63	7.46	0.190	0.607
GSH-Px (U/mg prot)	0.67 ^b^	1.15 ^a^	0.86 ^ab^	0.82 ^b^	0.054	0.003
T-AOC (mmol/mg prot)	43.61	47.34	47.24	50.53	0.866	0.067
MDA (nmol/mg prot)	0.20	0.13	0.14	0.16	0.024	0.737

Abbreviations—CON (control group): basal diet; BS (*Bacillus subtilis* group): basic diet + 5 × 10^9^ CFU/kg *Bacillus subtilis*; RYC (*Rhodotorula* yeast culture group): basic diet + 5000 mg/kg *Rhodotorula* yeast culture; MIX (mixture group): a basic diet + 5 × 10^9^ CFU/kg *Bacillus subtilis* + 5000 mg/kg *Rhodotorula* yeast culture; ^a,b^ Means within a row with different superscripts differ significantly (*p* < 0.05).

## Data Availability

The original contributions presented in this study are included in the article material.
